# Three-dimensional soft tissue landmark detection with marching cube algorithm

**DOI:** 10.1038/s41598-023-28792-w

**Published:** 2023-01-27

**Authors:** Yoonjung Lee, Ji-Min Lee, Sun-Hyung Park, Yoon Jeong Choi, Sung-Hwan Choi, Jae Joon Hwang, Hyung-Seog Yu

**Affiliations:** 1 Department of Orthodontics, Yonsei University College of Dentistry, 50-1 Yonsei-ro, Seodaemun-gu, Seoul, 03722 Republic of Korea; 2 Institute of Craniofacial Deformity, Yonsei University College of Dentistry, 50-1 Yonsei-ro, Seodaemun-gu, Seoul, 03722 Republic of Korea; 3 Department of Oral and Maxillofacial Radiology, School of Dentistry, Dental Research Institute, Pusan National University, Yangsan, 50610 Republic of Korea

**Keywords:** Orthodontics, Cone-beam computed tomography, Digital radiography in dentistry

## Abstract

Current method of analyzing three-dimensional soft tissue data, especially in the frontal view, is subjective and has poor reliability. To overcome this limitation, the present study aimed to introduce a new method of analyzing soft tissue data reconstructed by marching cube algorithm (Program S) and compare it with a commercially available program (Program A). Cone-beam computed tomography images of 42 patients were included. Two orthodontists digitized six landmarks (pronasale, columella, upper and lower lip, right and left cheek) twice using both programs in two-week intervals, and the reliability was compared. Furthermore, computer-calculated point (CC point) was developed to evaluate whether human error could be reduced. The results showed that the intra- and inter-examiner reliability of Program S (99.7–100% and 99.9–100%, respectively) were higher than that of Program A (64.0–99.9% and 76.1–99.9%, respectively). Moreover, the inter-examiner difference of coordinate values and distances for all six landmarks in Program S was lower than Program A. Lastly, CC point was provided as a consistent single point. Therefore, it was validated that this new methodology can increase the intra- and inter-examiner reliability of soft tissue landmark digitation and CC point can be used as a landmark to reduce human error.

## Introduction

Successful orthodontic treatment is defined as satisfying both patients and orthodontists by establishing a treatment plan based on a soft-tissue paradigm^1–5^. Achieving this requires a predictable visualized treatment objective (VTO) based on objective data. The existing VTO approach utilizing a two-dimensional (2D) lateral cephalogram cannot acquire three-dimensional (3D) facial soft tissue data. Thus, exact analysis and evaluation of 3D soft tissue from the frontal view is not possible. With the recent development of 3D imaging tools and software, many studies have been conducted on 3D soft tissue analysis. Pre-treatment VTO based on 3D facial analysis of pre- and post-treatment data is emerging as a field of interest in orthodontics and oral and maxillofacial surgery^6–8^.

Cone-beam computed tomography (CBCT) is widely used to gather both hard and soft tissue data. Facial soft tissue data acquired by CBCT can be processed via various commercially available programs such as INVIVO^6,7^, DOLPHIN 3D^9^, and MIMICS^10^. Numerous studies on 3D soft tissue analysis have been reported, such as digitizing 3D landmarks using various software, and comparing 3D landmarks and soft-tissue volume measurements before and after treatment^6–8^. However, in previous comparative studies on 3D soft tissue analysis, there was no perfect agreement between or within examiners^10,11^. This limitation is because existing dental software programs have mainly used volume-rendering techniques to express both soft and hard tissues^12^. This technique defines the color and transparency of all voxels of a 3D face and then projects it in 2D to express the detailed 3D shape of the face. However, it is difficult to acquire a reproducible point because volume-rendered images lack the ability to show the most protruded landmarks thoroughly, and the most superficial layer changes when the brightness and contrast are adjusted.

Owing to insufficient criteria and invalid methods of digitizing soft tissue landmarks, the present study applied the marching cube algorithm for the reconstruction of soft tissue data. The marching cube algorithm is a high-resolution 3D surface-rendering method widely used in the medical field, owing to its simplicity of implementation and relatively fast reconstruction^13^. By using the marching cube algorithm, 3D soft tissue images are reconstructed to show certain contour lines, and because of these contour lines, clinicians can intuitively see the most protruded area. Digitizing 3D soft tissue landmarks via contour lines seems to reduce the errors that occur in previously existing software programs. These errors included soft tissue appearing differently when the volume-rendered image was rotated or when the contrast, opacity, or brightness was manipulated. Therefore, we hypothesized that using the marching cube algorithm would increase the reliability of digitizing soft tissue landmarks.

If soft tissue analysis is performed using a 2D computer monitor for 3D structures, the lack of experience of orthodontists and surgeons accustomed to 2D diagnosis can affect the analyses of the results. To reduce and solve human errors in 3D data analysis, evaluating landmarks by employing a pre-educated artificial intelligence program or computer-aided analyzer might be recommended. These study results have led to the introduction of programs such as the YOLOv3 algorithm^14,15^ and WEBCEPH^16^. However, these programs are being used to measure 2D lateral cephalometric radiographs, and computer-aided 3D soft-tissue diagnostic methods are still insufficient.

The aim of this study was to introduce a method to analyze 3D CBCT soft tissue data reconstructed using the marching cube algorithm and compare it with landmarks digitized with the current commercially available software program. The null hypothesis was that there were no differences in reliability between the two programs. Moreover, we further evaluated whether human error could be reduced by proposing a specific computer-aided landmark named the computer-calculated point (CC point).

## Materials and methods

### Ethics statement

This study protocol was conducted with the approval of the Institutional Review Board (IRB) of Pusan National University Dental Hospital (IRB No.: PNUDH-2020-033) and was performed in accordance with the Declaration of Helsinki and relevant guidelines and regulations. This study passed the exemption review for informed consent on the use of patients’ CBCT images and medical records.

### Subjects

This study was conducted using CBCT images of patients at Pusan National University Dental Hospital from January 2019 to May 2022. Patients who were diagnosed with impaction of wisdom teeth or temporomandibular disorder without condylar bone changes were selected for this study. CBCT (Viso G7; Planmeca Oy, Helsinki, Finland) was performed with the following scanning parameters: 120 kVp, 11 mA, 36 s, voxel size of 0.3 mm, and field of view (FOV) of 16 × 16 cm. Images without blurring, severe metal artifacts, or surgical defects were selected. Images of patients whose eyelids to the tip of the chin were not captured and those whose nose or lips were cut from the image were excluded from the study.

The sample size was calculated using G * Power 3.1.9.7. (G * Power, version 3; Heinrich Heine University, Düsseldorf, Germany) with a *p* value < 0.05 indicating statistical significance, a power of 80%, and an effect size of 0.5 for detecting differences between programs^17^. The minimum sample size of 34 was required, and the present study included 42 participants.

### Methods

To perform the following process, we used a software using MATLAB (MathWorks, Matick, MA; 2022) for algorithm development, data analysis, visualization, and numerical calculation.

#### Soft tissue binarization

**Figure 1:**
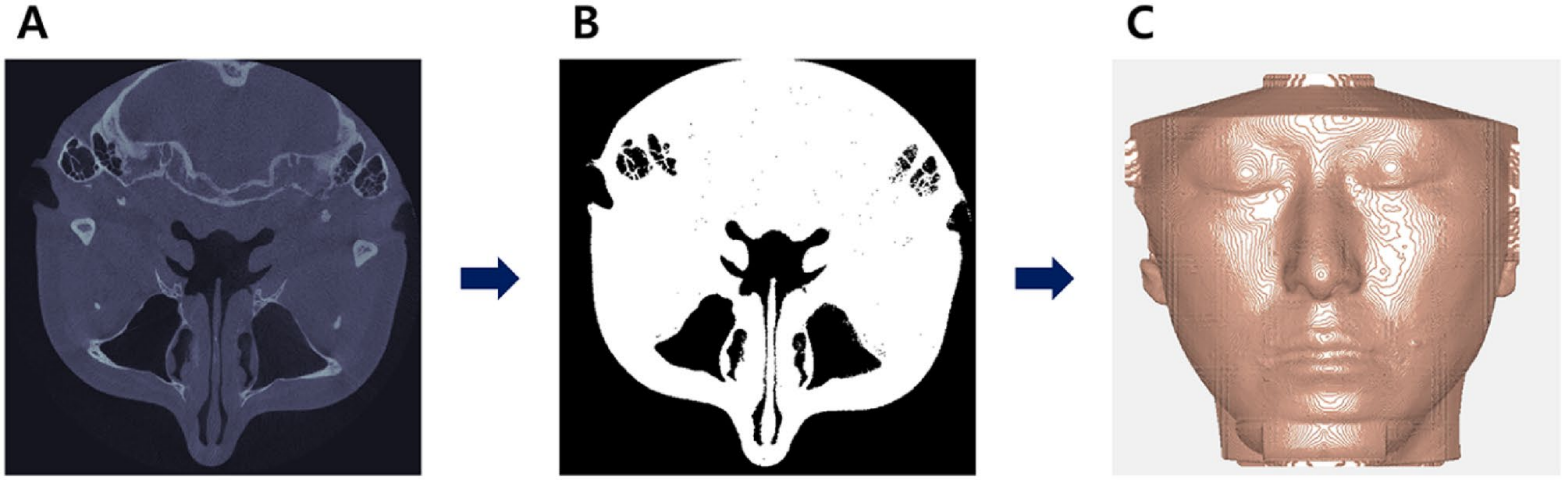
Image processing sequence. (**A**) Axial view of cone-beam computed tomography image. Hard tissues appear white, and soft tissues appear black or gray. (**B**) Automatic binarization using the Otsu method. Soft tissue images appear white. (**C**) Surface reconstruction using the marching cube algorithm. Contour lines are formed.

Automatic binarization of soft tissue was performed on 42 CBCT images using the Otsu method^18^ (Fig. 1). As a result of binarization, the soft and hard tissue images were inverted, and the soft tissue image could thus be obtained after separation.

#### Contour line formation using marching cube algorithm

The 3D surface reconstruction was done using the marching cube algorithm to form contour lines (Fig. 1C). A total of 42 files with enhanced soft tissue information in the the binary MATLAB files (.mat) were created using the soft tissue information obtained from the patient’s CBCT.

By using the marching cube algorithm, since CBCT images used in this experiment had a spatial resolution of 0.3 mm with an FOV of 16 × 16 cm, the soft tissue data was reconstructed to have stepped contour lines and mountain tabletop effects. However, the reconstruction process caused block noise; this was resolved by simplifying the entire surface and the contour lines and converging the vector direction of the soft tissue landmark to simple values.

It was confirmed that the normal vector on the contours of the protruded soft tissue points with a convex shape, as shown in Table 1, had a constant value between patients. This is because the anatomical definitions of these points correspond to the direction of the normal vector. That is, because the pronasale and both lip points are the most protruding points, they have a normal vector in the forward direction, and the columella and cheek points are located at the midpoint of the curved surface; thus, they have a normal vector inclined at 45° downward and laterally, respectively.

**Table 1:** Definition and normal vectors of soft tissue protrusion points.

Landmark	Abbreviation	Definition	Normal vector		
			x	y	z
Pronasale	Pn	The most anterior point of the nasal tip	0	− 0.5	0
Columella	Co	The most prominent point of the columella crest on the base view of the nose	0	− 0.5	− 0.5
Upper lip point	ULP	The most anterior point of upper lip	0	− 0.5	0
Lower lip point	LLP	The most anterior point of lower lip	0	− 0.5	0
Cheek_right	Ch_r	The most prominent point of the right cheek	− 0.5	− 0.5	0
Cheek_left	Ch_l	The most prominent point of the left cheek	0.5	− 0.5	0

#### Manual coordinate determination of soft tissue landmarks

According to the definition in Table 1, two orthodontists manually digitized six landmarks twice in two-week intervals on the center of the tabletop surrounding the protruded soft tissue points using current commercialized software [Program A (INVIVO6 software, version 6.5.0, Anatomage, San Jose, CA)] and homemade software using the marching cube algorithm (Program S).

Unlike in previous studies, the (0, 0, 0) reference point or reference plane was not set separately^6,9,19^. This was a method for reducing the error caused by setting the reference point and plane according to the examiners. The 3D coordinates were measured as (x, y, z), and Euclidean distance (d) between the coordinates was calculated and recorded according to the conventional method.

In both programs, positive values were indicated as left on the x-axis, backward on the y-axis, and upward on the z-axis. The 3D coordinate values expressed by the two programs were different. Therefore, for comparison, coordinates obtained from Program S were converted to the coordinate system of Program A by subtracting 266.5 for x, y coordinates and subtracting 266 for z coordinates, then multiplying each by 0.3.

**Figure 2:**
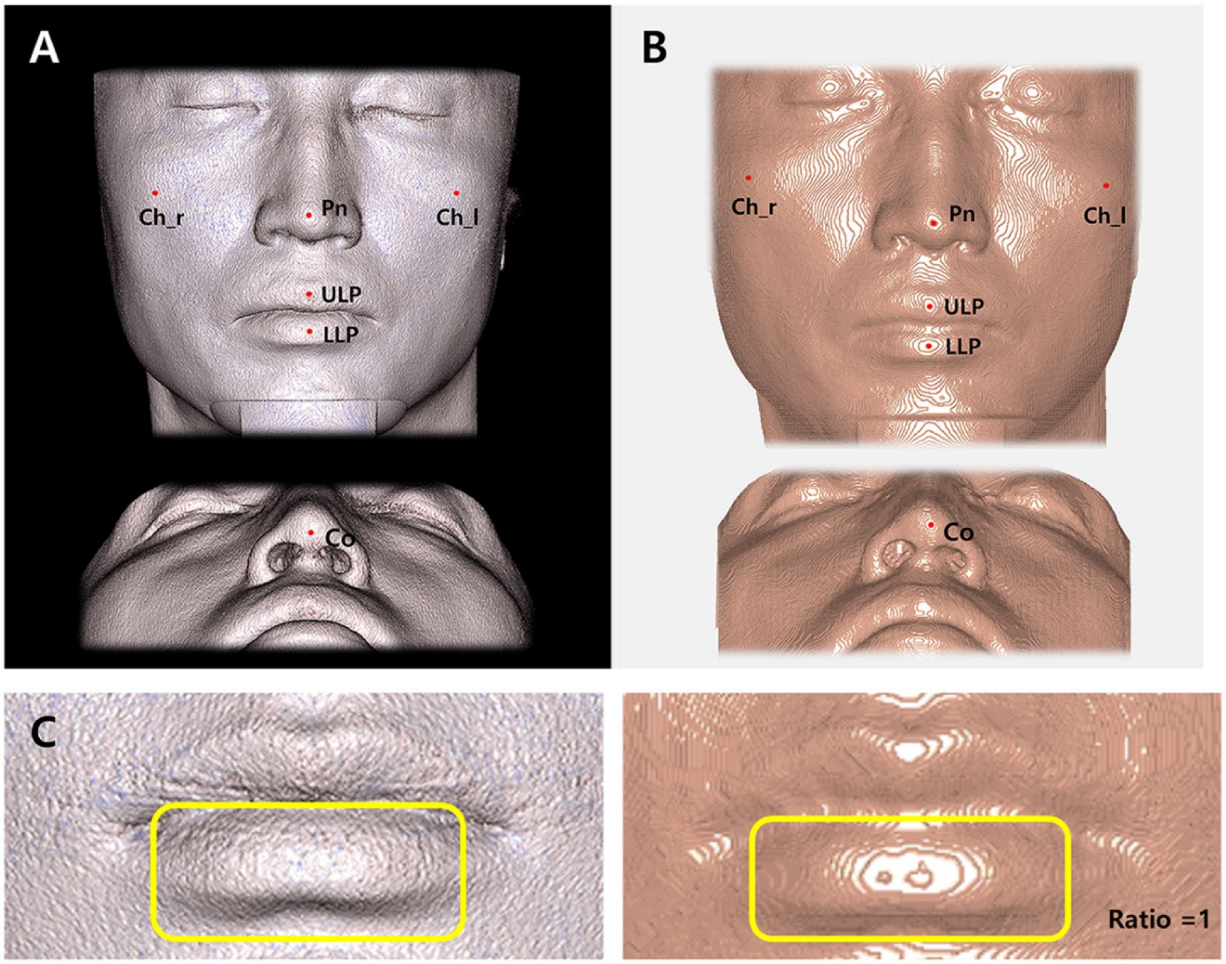
Schematic illustration of the landmarks used in this study. (**A**) Landmarks using the INVIVO6 software (Program A). (**B**) Landmarks using new homemade software (Program S). (**C**) Different lower lip images in the same patient (sample no. 023) according to program type. In Program A, detailed lower lip contour could not be observed. In Program S, contour lines with two tabletops with the same y-axis values were observed. The two most protruding parts could be accurately identified. Pn, Pronasale; Co, Columella; ULP, Upper lip point; LLP, Lower lip point; Ch_r, Cheek_right; Ch_l, Cheek_left.

The soft tissue landmarks used in this study were those that protruded out of soft tissue structures. As shown in Table 1, six landmarks were digitized manually at the center of each tabletop (Fig. 2).

#### Obtaining the CC point of soft tissue landmarks

**Figure 3:**
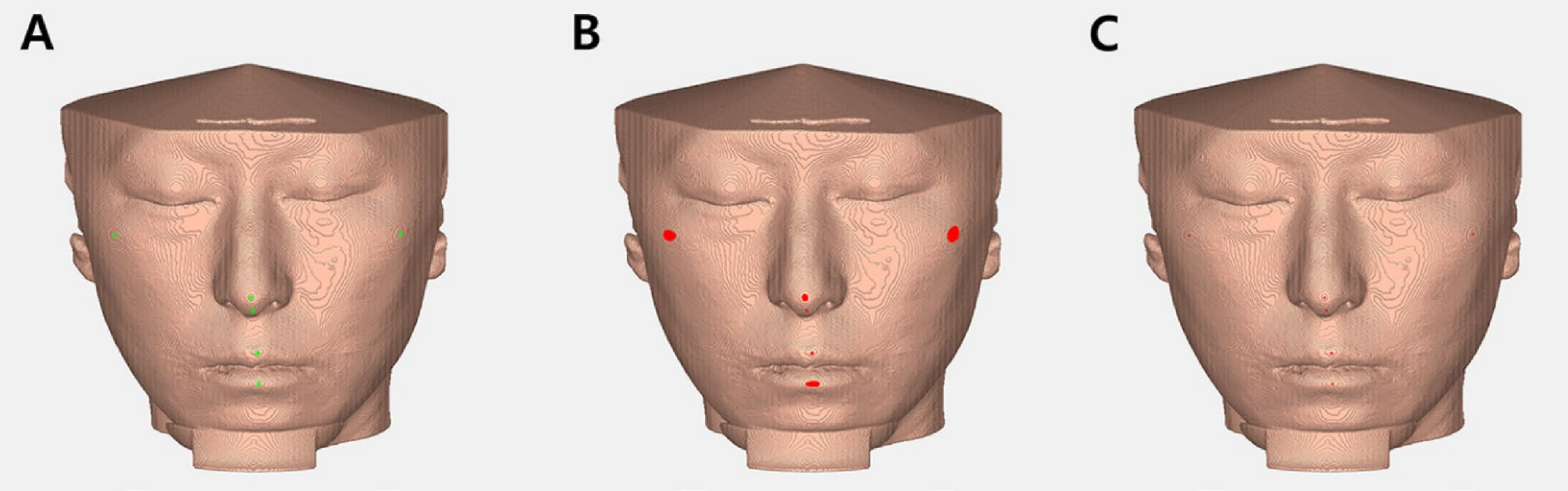
The process of obtaining the CC points of all the soft tissue landmarks (Full face view). (**A**) Manual coordinate determination of six landmarks (green dots). (**B**) Tabletop area (red area) including the coordinates of the starting points taken manually. (**C**) Average coordinates of the points (CC point, red dots) on the tabletop plane obtained.

**Figure 4:**
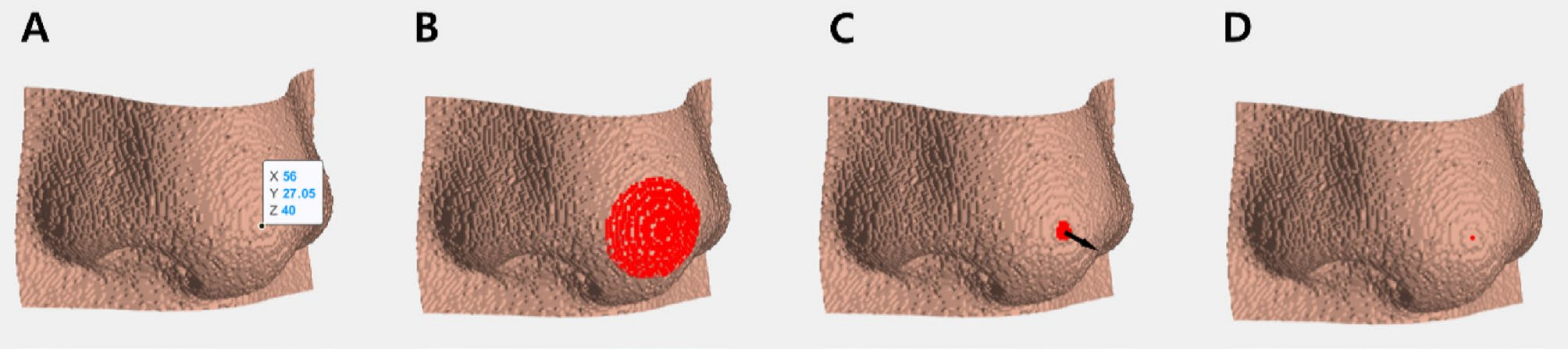
The process of obtaining the CC points of pronasale (left-sided view). (**A**) Manual coordinate determination of pronasale. (**B**) From the points taken manually, the points within 50 pixels of Euclidean distance were first obtained. (**C**) Tabletop plane including the coordinates of the points taken manually representing normal vector (black arrow). (**D**) Average coordinates of the points on the tabletop plane obtained.

Because the landmarks were digitized manually, even if they were digitized meticulously to the center of the tabletop as closely as possible, human errors might still occur. Therefore, we developed an algorithm to automatically find all the coordinates making up the tabletop and determine the exact center point of protruded soft tissue landmarks using the center coordinate of the tabletop. The points determined by this method were called the computer-calculated (CC) points. The detailed method for obtaining the CC point is as follows:

1. Obtaining the tabletop plane containing the starting point: From the starting points obtained manually (Fig. 3A, 4A), points within 50 pixels of Euclidean distance were first obtained (Fig. 3B, 4B). Among them, the points within one pixel from the starting points in the direction of the normal vector, referring to Table 1, remained (Fig. 4C).
2. Obtaining the CC point: The average coordinates of the points on the tabletop obtained in step 1) (Fig. 3C, 4D) were determined as the CC points.

Using the 42 samples, the CC points (CC1, CC2) of each examiner were calculated using the measurements acquired in Program S, and the degree of agreement between CC1 and CC2 was investigated using Cronbach’s alpha coefficient.

#### Consistency examination of CC point

**Figure 5:**
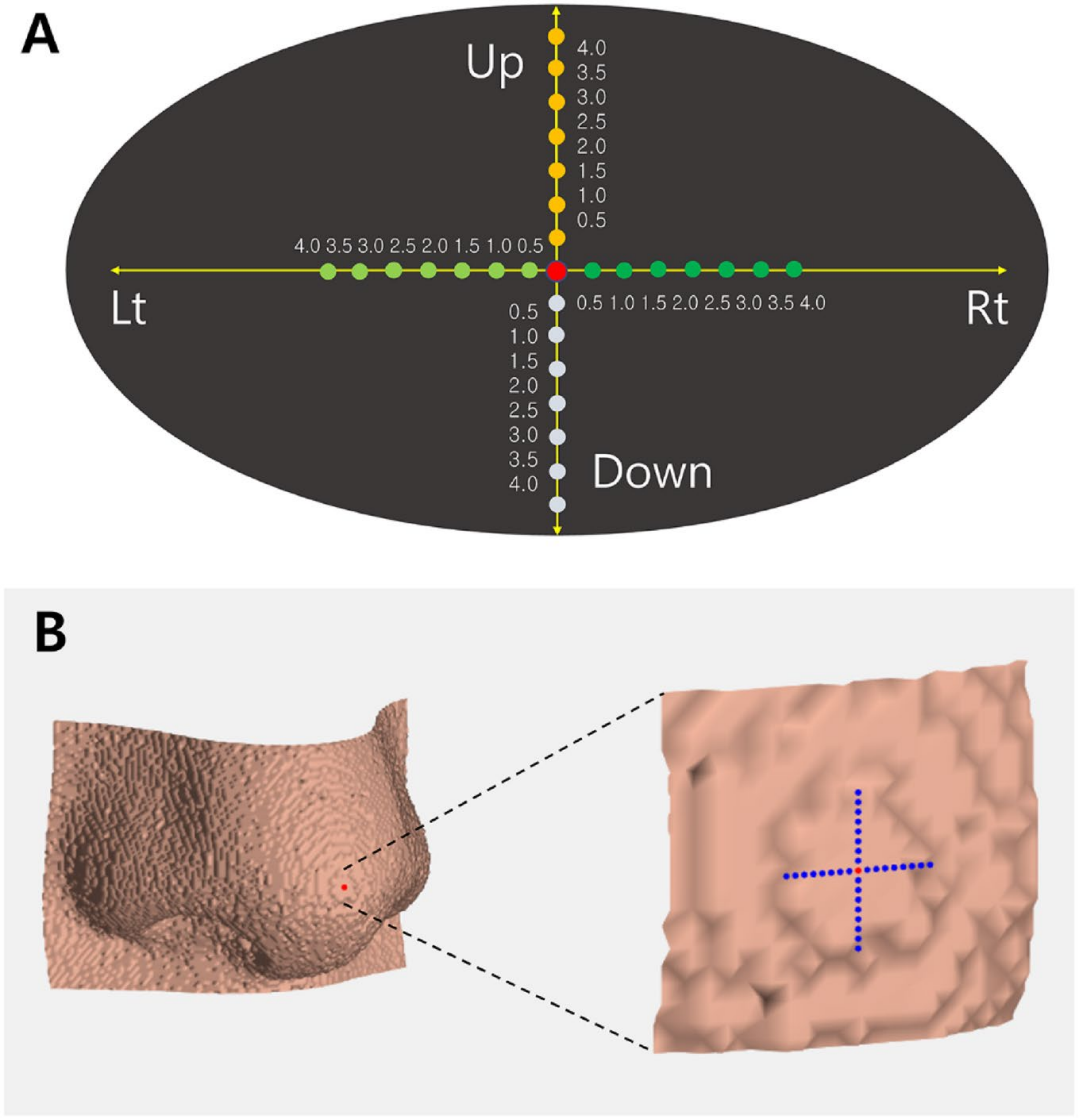
Consistency examination of CC point. (**A**) The distance between the original coordinate and second coordinate that was moved 0.5 pixels in each direction point (yellow, light green, green, and white dots) was compared; red dot, original coordinate; yellow arrow, movement direction; Up, upward; Down, downward; Rt, rightward; Lt, leftward. (**B**) Example for pronasale; the red dot was an original CC point; blue dots are coordinates that were moved by 0.5, 1.0, 1.5, 2.0, 2.5, 3.0, 3.5, and 4 pixels in the up, down, left, and right directions.

To confirm whether the points that comprise the tabletop consistently refer to the same CC point, new CC points were created and compared to the original point using the following method. After moving the points by 0.5, 1.0, 1.5, 2.0, 2.5, 3.0, 3.5, and 4 pixels in the up, down, left, and right directions from the CC point (Fig. 5), the second CC points were obtained from the moved coordinates as input. The consistency of the developed algorithm was verified, and its clinical validity was evaluated by calculating the distance (Δd) between the original and the second CC point.

### Statistical analysis

Statistical analyses were performed using PASW Statistics 18 (SPSS, Chicago, IL). The 95% confidence level (*p* < 0.05) was considered statistically significant. The reproducibility of digitizing the landmarks at a two-week interval was assessed by each examiner (intra-examiner). Additionally, the reproducibility of landmarks was assessed between the two examiners using each program (inter-examiner). Cronbach’s alpha coefficient was used to determine the intra- and interclass correlation coefficients. The difference (Δx, Δy, Δz, Δd) in the measured values between the two examiners (Ob1 and Ob2) according to each program was calculated. A paired *t*-test was performed to determine whether there was a significant difference in precision between Programs A and S among the operators for the six landmarks. Additionally, Cronbach’s alpha coefficient was calculated to evaluate the reliability of CC1 and CC2.

## Results

The intraclass correlation coefficient was statistically analyzed for differences between each examiner’s first and second trials in measured values of each of the x, y, and z coordinates and distance (Table 2). In Program A, Cronbach’s alpha coefficient of each examiner ranged from 64.0 to 99.9%. For the four landmarks (Pn, Co, ULP, and LLP), reliability was > 90%. However, for Ch_r (64.0%) and Ch_l (76.2%) at the x component by examiner 1, the reliability was < 80%. In Program S, Cronbach’s alpha coefficient of each examiner was between 99.7 and 100%, meaning that each examiner’s first and second trials were almost identical, which was statistically significant.

**Table 2:** The difference in intra-examiner reliability between Program A and Program S.

Landmarks	Program A		Program S	
	Ob 1	Ob 2	Ob 1	Ob 2
Pn				
x component	0.992	0.986	0.997	0.999
y component	0.987	0.998	1.000	1.000
z component	0.997	0.997	1.000	1.000
Distance	0.987	0.998	1.000	1.000
Co				
x component	0.995	0.980	0.997	0.999
y component	0.945	0.865	0.998	1.000
z component	0.996	0.992	1.000	1.000
Distance	0.953	0.877	1.000	1.000
ULP				
x component	0.968	0.973	0.997	0.998
y component	0.996	0.998	1.000	1.000
z component	0.996	0.993	1.000	1.000
Distance	0.997	0.996	0.999	0.999
LLP				
x component	0.947	0.962	0.998	0.999
y component	0.999	0.999	1.000	1.000
z component	0.995	0.995	1.000	1.000
Distance	0.997	0.995	0.999	0.999
Ch_r				
x component	0.640	0.839	0.999	1.000
y component	0.829	0.940	0.999	1.000
z component	0.965	0.953	1.000	1.000
Distance	0.918	0.949	1.000	1.000
Ch_l				
x component	0.762	0.831	0.999	1.000
y component	0.869	0.929	0.999	1.000
z component	0.957	0.963	1.000	1.000
Distance	0.963	0.958	1.000	1.000

Cronbach’s alpha coefficient was obtained to assess the intraclass correlation coefficient. Pn, Pronasale; Co, Columella; ULP, Upper lip point; LLP, Lower lip point; Ch_r, Cheek right; Ch_l, Cheek left; Ob 1, examiner 1; Ob 2, examiner 2.

Because the intraclass correlation coefficient value was reliable, the average values of the first and second trials were calculated for each examiner and used as a representative value of each examiner to analyze the interclass correlation coefficient (Table 3). In Program A, the reliability of the four landmarks (Pn, Co, ULP, and LLP) showed a high degree of agreement (> 90%). However, the reliability of the Ch_r and Ch_l points was < 90% (right cheek, 76.1%; left cheek, 88.2%). In Program S, the reliability was 99.9% to 100%, indicating almost perfect agreement between the two examiners.

**Table 3:** The difference in inter-examiner reliability between Program A and Program S.

Landmarks	Program A	Program S
Pn		
x component	0.991	0.999
y component	0.996	1.000
z component	0.998	1.000
Distance	0.996	1.000
Co		
x component	0.993	0.999
y component	0.972	1.000
z component	0.998	1.000
Distance	0.976	1.000
ULP		
x component	0.983	0.999
y component	0.998	1.000
z component	0.996	1.000
Distance	0.998	1.000
LLP		
x component	0.955	1.000
y component	0.999	1.000
z component	0.996	1.000
Distance	0.997	1.000
Ch_r		
x component	0.761	1.000
y component	0.922	1.000
z component	0.972	1.000
Distance	0.954	1.000
Ch_l		
x component	0.882	1.000
y component	0.944	1.000
z component	0.971	1.000
Distance	0.980	1.000

Cronbach’s alpha coefficient was obtained to assess the intraclass correlation coefficient. Pn, Pronasale; Co, Columella; ULP, Upper lip point; LLP, Lower lip point; Ch_r, Cheek right; Ch_l, Cheek_left.

To evaluate the difference in the precision of digitizing the landmarks, the difference in the coordinates (Δx, Δy, Δz) and distance (Δd) between the two examiners according to each program was calculated (Table 4). The difference of the coordinates and the distance of all six landmarks using Program S were less than those using Program A (*p* < 0.05). For Ch_r and Ch_l, the difference in the coordinates was greater than 1.0, but less than 0.1 in Program S (*p* < 0.001).

**Table 4:** Comparison of differences in measurement values (Δx, Δy, Δz, Δd) between the two examiners.

Landmarks	Program A		Program S		*p* value
	Mean	SD	Mean	SD	
Pn					
Δx	0.305	0.276	0.053	0.086	< 0.001*
Δy	0.117	0.237	0.000	0.001	0.003*
Δz	0.532	0.478	0.089	0.105	< 0.001*
Δd	0.128	0.238	0.007	0.009	0.002*
Co					
Δx	0.294	0.221	0.086	0.095	< 0.001*
Δy	0.599	0.472	0.063	0.078	< 0.001*
Δz	0.506	0.396	0.063	0.078	< 0.001*
Δd	0.545	0.446	0.056	0.067	< 0.001*
ULP					
Δx	0.416	0.293	0.089	0.105	< 0.001*
Δy	0.171	0.192	0.000	0.000	< 0.001*
Δz	0.668	0.509	0.086	0.095	< 0.001*
Δd	0.246	0.250	0.031	0.034	< 0.001*
LLP					
Δx	0.663	0.527	0.068	0.089	< 0.001*
Δy	0.136	0.147	0.004	0.024	< 0.001*
Δz	0.954	0.598	0.093	0.099	< 0.001*
Δd	0.470	0.337	0.047	0.048	< 0.001*
Ch_r					
Δx	4.813	2.770	0.062	0.079	< 0.001*
Δy	3.151	1.948	0.058	0.078	< 0.001*
Δz	3.192	2.271	0.118	0.126	< 0.001*
Δd	1.196	0.875	0.022	0.026	< 0.001*
Ch_l					
Δx	3.165	2.251	0.04	0.064	< 0.001*
Δy	2.182	1.624	0.04	0.064	< 0.001*
Δz	3.295	2.166	0.143	0.115	< 0.001*
Δd	0.808	0.629	0.028	0.03	< 0.001*

Data are presented as* p* value calculated with paired t-test between differences in measurement values (Δx, Δy, Δz, Δd (mm)) between examiner 1 and 2 when measured using each program. Pn, Pronasale; Co, Columella; ULP, Upper lip point; LLP, Lower lip point; Ch_r, Cheek right; Ch_l, Cheek_left. SD, standard deviation; *p value < 0.05.

**Figure 6:**
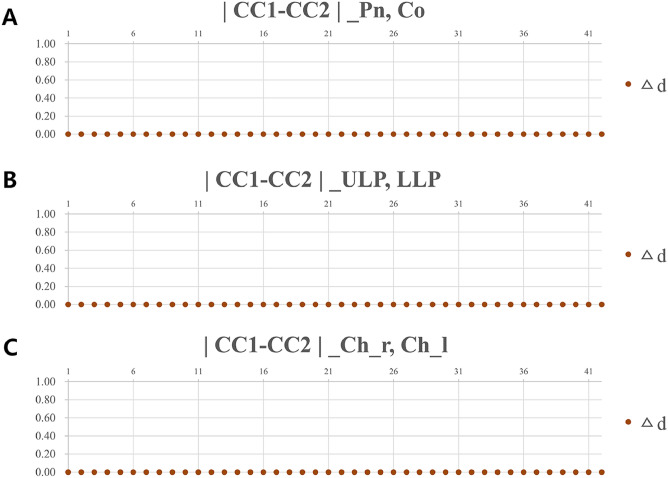
The differences in CC points of the 3D soft tissue landmarks using 42 samples derived from each examiner. (**A**) Pronasale and columella. (**B**) Upper lip and lower lip. (**C**) Cheek right and cheek left. CC1, CC point from examiner 1; CC2, CC point from examiner 2; Δd, difference of distances between CC1 and CC2.

The concordance between the measured values of CC1 and CC2 was investigated by calculating the difference arithmetically. The difference of distances (Δd) between CC1 and CC2 for all six landmarks of the 42 images were zero (Fig. 6), and Cronbach’s alpha coefficient between CC1 and CC2 showed a value of 1 for all the landmarks. The CC points measured and calculated using Program S were 100% consistent for all six landmarks.

The distance between the original CC point and the CC point derived by moving from 0 to 4 pixels (second CC point) was evaluated. All distance values were zero pixels.

## Discussion

The present study showed that the marching cube algorithm for soft tissue landmark detection had high intra- and inter-examiner reliability. Moreover, a newly proposed coordinate, the CC point, was more consistent than manual digitation, and was thus validated as a more consistent and reliable landmark in clinical practice.

**Figure 7:**
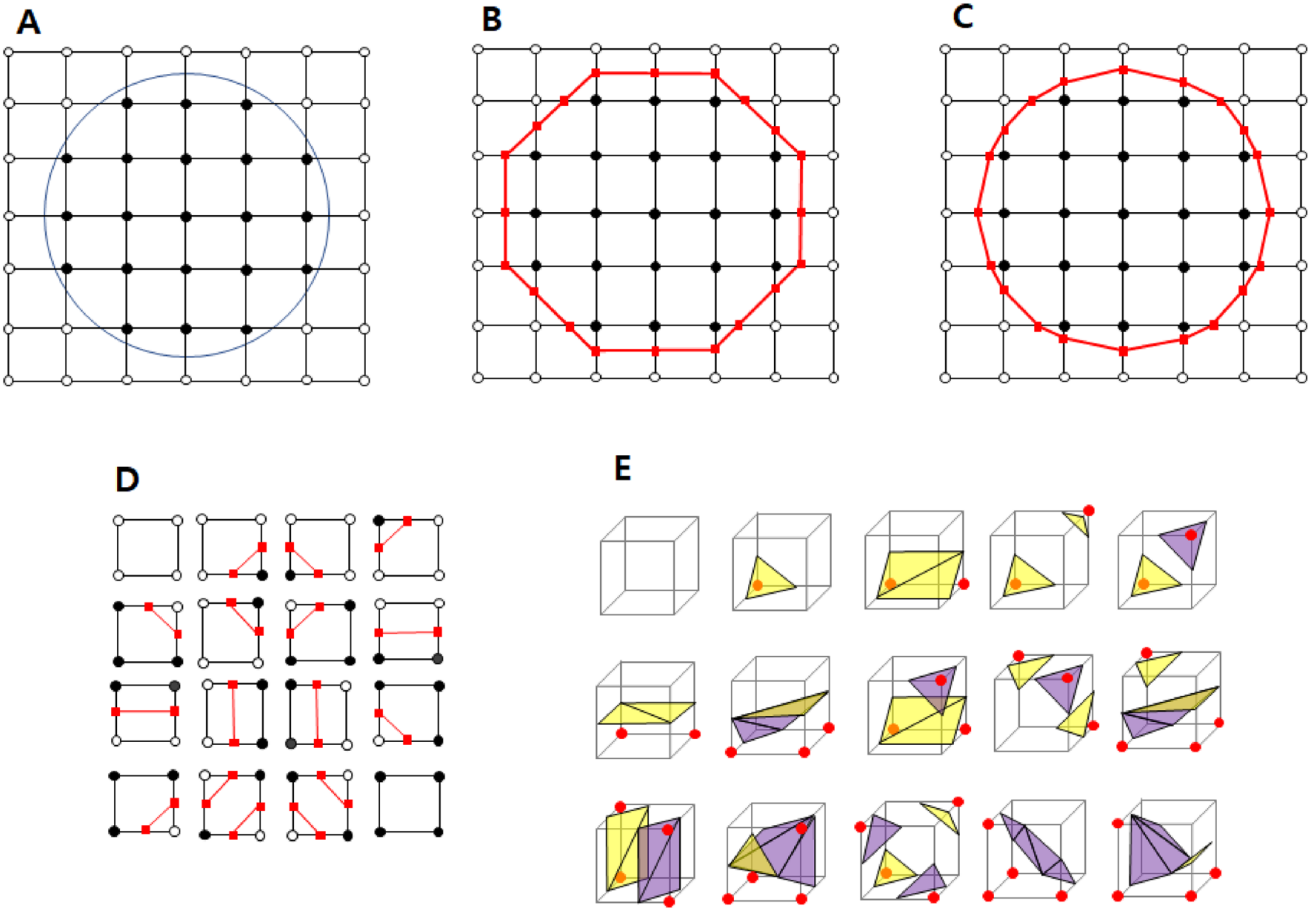
Marching cube algorithm. (**A**) The algorithm divides the space into uniform cell units and divides the cells in and out of the circle. (**B**) Boundary making matching the dictionary of 16 possible cases. (**C**) Distance from the center to the edge is approximated to the actual distance. (**D**) All 16 2D marching cube combinations used to make the boundary. (**E**) All 15 3D marching cube combinations used to make the boundary.

The marching cube algorithm was used in Program S to reconstruct the soft tissue data and form contour lines and tabletops to facilitate the digitization of the soft landmarks. The principle for the formation of contour lines via the marching cube algorithm is as follows. In 2D space, the marching cube algorithm divides the space into uniform cell units and divides the cells in and out of the circle (Fig. 7A). Boundaries can quickly be drawn while matching the dictionaries of the 16 possible cases (Fig. 7B). Subsequently, the distance from the center to the edge was approximated as the actual distance (Fig. 7C). All 16 2D marching cubes combinations are used to make the boundaries (Fig. 7D). In 3D space, this algorithm uses a cube as a unit to form an isosurface. The cube configurations formed during the triangulation step can generally be categorized into 15 unique patterns identified in the original marching cube algorithm (Fig. 7E)^20^. Therefore, the marching cube algorithm independently selects 1 of 15 patterns in each cell, enabling parallel processing, and then processes single-image results on a unique surface.

The degree of smoothing of the contours made by the marching cube algorithm is affected by the resolution of the 3D image: the higher the resolution, the smaller the size of the cube making up the surface, and as a result, more detailed surface images and dense contours can be obtained. Therefore, a smooth surface in which contours are not clearly visible is obtained after surface reconstruction using a marching cube algorithm, such as an intraoral scanner, for equipment with high spatial resolution. The CBCT images used in this experiment had a spatial resolution of 0.3 mm with an FOV of 16 × 16 cm, and this relatively low spatial resolution caused stepped contours and mountain tabletop effects to be generated during reconstruction with the marching cube algorithm. The result of these different resolutions is clearly visible in the marching cube results of the CBCT images with different resolutions.

Therefore, by this process, Program S showed a slight difference in the height of the soft tissue as a contour line and served as a guide when digitizing landmarks manually. Digitizing 3D soft tissue landmarks via contour lines reduces errors when evaluating 3D structures via 2D screens and resolves issues with soft tissue appearing differently when the rendered images are rotated or when the contrast, opacity, or brightness are manipulated. As a result, the intra- and interclass correlation coefficients were close to 1, indicating that the exact same point could be measured multiple times. Moreover, the difference in the measurement values between examiners (Δx, Δy, Δz, Δd) for Program S was less than that for Program A, and the differences were close to zero. In other words, the precision of the landmarks between examiners was higher when using Program S compared to Program A. Furthermore, in the present study, by calculating the average coordinates of the points on the tabletop, the CC point of the 3D soft tissue was obtained. There was no difference in the landmarks depending on the acquisition time and examiners, and it was possible to obtain completely consistent measurements. That is, it was possible to digitize the 3D soft tissue landmarks more reliably and objectively using Program S and CC points (especially cheek points on both sides). Since orthodontic and surgical treatments are mostly irreversible, treatment plans should be established not just by instinct, but with accuracy. In this study, we proposed a methodology for digitizing soft tissue landmarks that is highly reliable; therefore, by using this methodology, more accurate research can be conducted regarding soft tissue changes before and after treatment.

Using Program S, detailed digitization was possible even on other soft tissue areas and not on specific landmark points. For example, in Sample 23, two high contour lines were observed on the lower lip (Fig. 3). This meant that the patient’s lips were slightly concave in the center. In Program A, it was difficult to observe these characteristics. Therefore, Program S can be used for delicate soft tissue changes caused by soft tissue fillers or botulinum toxin.

Various methods, such as 3D-stereophotogrammetry^19,21^, Moire topography^22^, optical surface scans^23^, laser scanning^24,25^, and CBCT scans^6,9,19^, have been introduced to acquire 3D facial soft tissue data. In this study, CBCT was used for soft tissue data acquisition because the subjects underwent CBCT taking for the extraction of wisdom teeth. Likewise, orthodontists and oral-maxillofacial surgeons mostly modify the hard tissues (skeletal and teeth) to acquire the desired soft tissue change. Therefore, CBCT is the most convenient choice for acquiring both hard and soft tissue data simultaneously. Moreover, a previous study showed that CBCT is the most efficient and reliable imaging tool for soft tissues^26^, and so using CBCT for soft tissue evaluation was a reasonable choice for this study. However, CBCT inevitably exposes patients to radiation^27^. Therefore, in the cosmetic field, the degree of skin shrinkage or skin elasticity is often evaluated using 3D-stereophotogrammetry^21^ and Moire topography methods^22^, limiting radiation exposure. If the clinician uses only soft tissue procedures, such as botulinum toxin or fillers, laser scanners are often used as a simple method^24,28^. Previous studies defined soft tissue landmarks based on the underlying hard tissue structures; consequently, if there is no hard tissue data, clinicians cannot detect soft tissue landmarks^29^. Moreover, soft tissue landmarks determined based on the underlying hard tissue are not always clinically meaningful, since as the soft tissue elasticity decreases or as the patients become more obese, the soft tissue becomes less associated with the underlying hard tissues^30–32^. This study provides a reliable method for digitizing soft tissue landmarks without the need for hard tissue. Therefore, this method can be applied to soft tissue acquisition methods other than CBCT. Further research on applying this method to other soft tissue acquisition methods will be conducted in the future, but similar results are expected as the marching cube algorithm can be applied to any data.

Until now, comparisons before and after treatment were inaccurate because there was a lack of studies that questioned the reliability of the current method of digitizing soft tissue landmarks. Therefore, most studies used 3D facial heatmaps to show changes in the soft tissue profile^33^. Using only heatmaps, clinicians are unable to determine the exact amount of soft tissue changes, leading to inaccurate treatment plans. Compared to heatmaps, digitizing landmarks and evaluating the change in these landmarks before and after treatment would allow clinicians to determine the exact changes.

In previous studies, most soft tissue landmarks were digitized on the lower facial area and along the midsagittal plane^6–8^. However, Ferrario et al.^35^ in actual clinical practice, meaningful soft tissue landmarks are not necessarily located along the midsagittal plane. In particular, there are not enough standards and evaluations on features of the cheek area, which occupies a large portion of the face. Soft tissue of the cheek area relies on the structure of the underlying hard tissue to a certain extent; however, features such as protrusion and inversion of the cheeks vary depending on muscle development, amount of subcutaneous fat, patients’ sex, age, and elasticity of the skin tissue^6^. As a result, the definition and method of measuring landmarks on the cheek area differed amongst each study. According to Menezes et al., it was defined as the intersection of Camper’s plane and a line connecting the external eye canthus with the labial commissure^34,35^. In another study, it was defined as the intersection of the vertical line passing through the mid-canthus parallel to the z-axis and the horizontal line passing through the ala and perpendicular to the vertical line^36^. This area did not protrude from the center of the cheek, nor was it in the middle of the cheek area; neither point was clinically meaningful. Hence, before-and-after evaluations of cheek area modifications such as MEDPOR augmentation^37^, soft tissue treatments with botulinum toxins^38–40^, malar-plastic surgery^41^, and changes in soft tissue when treated by mini-screw-assisted rapid palatal expanders^36^ could not be precisely assessed using these landmarks. However, in this study, when using the marching cube algorithm to digitize dots on the cheeks (Ch_r, Ch_l) using Program S, the most protruding area could be evaluated, which is clinically significant, and can possibly be applied to the evaluation of the forementioned treatments.

The developed algorithm can be applied not only to the field of medical imaging, but also to various studies that require reference points on various curved surfaces, such as animation, 3D land or remains surveys, and nanosurface measurements. The marching cube algorithm produces a mesh that is a rough approximation of the true isosurface and is not well suited to sharp corners or creases^42^. However, the developed algorithm was implemented on a soft, relatively simple face-shaped image with low enough resolution to show the mountain tabletops and contour lines, which is an acceptable use of the marching cube algorithm. Since mountain tabletops, which are the result of the marching cube algorithm, are objects that can be easily recognized visually, it is expected that the entire process can be fully automated through automatic segmentation using deep learning in the future.

There are a few limitations of this study. First, due to the scattered radiation, soft tissue data acquired via CBCT have low contrast resolution^43^. Despite the convenience of CBCT, applying any algorithm to reconstruct the CBCT soft tissue data has risks of analyzing inaccurate data. However, previous studies indicated that there were no clinical differences between other types of soft tissue acquisition methods^11,44^; therefore, the results of this study could be considered acceptable. Nevertheless, future studies are needed to determine whether similar results are obtained for other acquisition methods. Second, only six landmarks were included in this study. More landmarks could have been included, such as soft tissue pogonion or glabella. However, in the process of immobilizing the patient during CBCT acquisition, deformation of the soft tissue pogonion and glabella was caused by the chin and forehead resting on the CBCT scanner. Therefore, based on the results of this study, future studies using CBCT data without deformation of the chin and forehead are required. In the case of soft tissue gonions, manual digitation by using Program S was also possible for most subjects. However, the soft tissue gonion was difficult to detect in subjects that lacked soft tissue elasticity. Moreover, because of the overlapping contour lines in the gonion area, it was difficult to develop the CC point using the current algorithm. Finally, only convex landmarks were evaluated in this study. Contour lines made by the marching cube algorithm made stable tabletops on convex points but not on concave points. An improved algorithm and methodology should be developed to overcome these limitations.

## Conclusion

A newly developed Program S, which reconstructs soft tissue data using the marching cube algorithm, allows clinicians to manually detect and digitize six soft tissue landmarks more reliably than currently existing software. Moreover, from the contour lines formed by the algorithm, the computer can automatically detect the CC point, which is the center of the most protruded area for each landmark, and is consistent and reliable. This new methodology for detecting soft tissue landmarks will help clinicians analyze soft tissue changes more objectively and help establish a treatment plan more accurately.

## Data Availability

The data underlying this article cannot be publicly shared to protect the privacy of the individuals participating in the study. The data will be shared at a reasonable request to the corresponding author.
