# Author Correction: Three-dimensional soft tissue landmark detection with marching cube algorithm

**DOI:** 10.1038/s41598-023-29751-1

**Published:** 2023-02-17

**Authors:** Yoonjung Lee, Ji-Min Lee, Sun-Hyung Park, Yoon Jeong Choi, Sung-Hwan Choi, Jae Joon Hwang, Hyung-Seog Yu

**Affiliations:** 1 Department of Orthodontics, Yonsei University College of Dentistry, 50-1 Yonsei-ro, Seodaemun-gu, Seoul, 03722 Republic of Korea; 2 Institute of Craniofacial Deformity, Yonsei University College of Dentistry, 50-1 Yonsei-ro, Seodaemun-gu, Seoul, 03722 Republic of Korea; 3 Department of Oral and Maxillofacial Radiology, School of Dentistry, Dental Research Institute, Pusan National University, Yangsan, 50610 Republic of Korea

Correction to: *Scientific Reports* 10.1038/s41598-023-28792-w, published online 27 January 2023

In the original version of this Article Yoonjung Lee and Ji-Min Lee were omitted as equally contributing authors.

The original Article has been corrected.

